# Reviewed article: Vale D, Andrade MEC, Dantas NM. Adherence to multiple recommendations of the Dietary Guidelines for the Brazilian Population (Guia Alimentar para a População Brasileira, GAPB) and associated factors among adolescents: a cross-sectional study with data from the National Students’ Health Survey, 2019. Epidemiol Serv Saude. 2025:34;e20240404.

**DOI:** 10.1590/S2237-96222025v34e20240404.a

**Published:** 2025-06-13

**Authors:** Larissa Fortunato Araújo

**Affiliations:** Universidade Federal do Ceara, saúde comunitária

**Table:** 

Rating	Excellent	Good	Average	Below average	Poor
Interest		✓			
Quality		✓			
Originality		✓			
Overall		✓			

**Table:** 

Questionnaire	Yes	No	Not applicable
Does the manuscript contain new and significant information to justify publication?	✓		
Does the Abstract (Summary) clearly and accurately describe the content of the article?	✓		
Is the problem significant and concisely stated?	✓		
Are the methods described comprehensively?	✓		
Are the interpretations and conclusions justified by the results?	✓		
Is adequate reference made to other work in the field?	✓		

## Manuscript Structure

Length of article is: Adequate

Number of tables is: Adequate

Number of figures is: Adequate

Please state any conflict(s) of interest that you have in relation to the review of this paper (state “none” if this is not applicable): None

## Comments

Estudo de temática relevante. Contudo, a descrição do métodos, principalmente, precisa ser melhor redigida. Além da necessidade de mais profundidade teórica.

Resumo:

Inclua as direções das associações.

Introdução:

- Senti falta na introdução e também de maior profundidade na discussão sobre a relação das variáveis explicativas com seu desfecho, estudos semelhantes, divergentes, lacunas....

Métodos:

- O desenho do estudo é ecológico? Misto?

- Como as suas duas variáveis, mais e menos restrito, chegou a u a pontuação de 0 a 10? O que vcs pontuaram como 0 em não deveria ser 1 ponto, não?

- Ainda não está claro como você criou a sua variável resposta (do modelo com ajustamento múltiplo) Como vc transformou ela em binária? Tudo relativo a categorização deveria ficar junto da descrição de criação da variável. Ademais qual criterio ou referência para a criação do seu desfecho?

- Por que você performou o modelo com ajustamento múltiplo com apenas um desfecho? Deixar toda a análise restrita apenas a ele?

- Você testou comparar em acima e abaixo da mediana para o modelo mais restrito?

Resultados:

- Tem alguma inconsistência na análise do Índice de Moran. Apresenta apenas um tom, por que não mostra Brasil? Vocês utilizaram qual informação para o georreferenciamento?

- Tabela 2 incluir o valor de p de todos os testes e modelos.

Discussão:

- Idealmente a discussão inicia com a retomada dos principais achados do estudo.

- Incluir em limitações a ausência de endereço do indivíduo. Imagino que vocês georreferenciaram o estado, certo? Isso pode ter impactado na analise de autocorrelação. Também, incluir possíveis vieses do desenho e variáveis.

- Incluir uma conclusão.

